# Comparable long‑term survival outcomes of endoscopic treatment versus surgical treatment for gastrointestinal stromal tumors with a diameter of 5–10 cm

**DOI:** 10.1038/s41598-024-58802-4

**Published:** 2024-04-12

**Authors:** Zide Liu, Qing Tao, Yonghui Wu, Chunyan Zeng, Youxiang Chen

**Affiliations:** 1 Department of Gastroenterology, Digestive Disease Hospital, The First Affliated Hospital, Jiangxi Medical College, Nanchang University, Nanchang, China; 2 Jiangxi Clinical Research Center for Gastroenterology, Nanchang, Jiangxi China

**Keywords:** Gastrointestinal stromal tumors, SEER database, Endoscopy, Surgery, Gastrointestinal diseases, Gastrointestinal cancer

## Abstract

Currently, endoscopic treatment for small gastrointestinal stromal tumors (GIST) has been widely accepted. However, for tumors larger than 5 cm, endoscopic treatment has not been recognized by national guidelines as the standard therapy due to concerns about safety and adverse tumor outcomes. Therefore, this study compares the long-term survival outcomes of endoscopic treatment and surgical treatment for GIST in the range of 5–10 cm. We selected patients with GIST from the Surveillance, Epidemiology, and End Results (SEER) database between 2004 and 2015. Kaplan–Meier analysis and the log-rank test were employed to compare the long-term survival outcomes between endoscopic treatment and surgical treatment. A multivariate Cox proportional hazards model was used for analysis to identify risk factors influencing patient prognosis. To balance baseline data, we performed 1:1 propensity score matching (PSM). A total of 1223 GIST patients were included, with 144 patients (11.8%) received endoscopic treatment and 1079 patients (88.2%) received surgical treatment. Before PSM, there was no significant difference in the long-term survival rates between the two groups [5-year OS (86.5% vs. 83.5%, *P* = 0.42), 10-year OS (70.4% vs. 66.7%, *P* = 0.42)]. After adjusting for covariates, we found that the overall survival (HR = 1.26, 95% CI 0.89–1.77, *P* = 0.19) and cancer-specific survival (HR = 1.69, 95% CI 0.99–2.89,* P* = 0.053) risks were comparable between the endoscopic treatment group and the surgical treatment group. In the analysis after PSM, there was no significant difference between the endoscopic treatment group and the surgical treatment group. Our study found that for GIST patients with tumor sizes between 5 and 10 cm, the long-term OS and CSS outcomes were similar between the endoscopic treatment group and the surgical treatment group.

## Introduction

Gastrointestinal stromal tumors (GIST) are the most common mesenchymal-derived tumors of the digestive tract, primarily originating from Cajal cells or their precursor cells^1^. The biological behavior of GIST is diverse, manifesting as benign, potentially malignant, or varying degrees of malignancy, the majority of GIST exhibit mutations in KIT or platelet derived growth factor receptor alpha (PDGFRA)^2,3^. The incidence of GIST ranges from 6 to 22 cases per 10^6^ individuals per year^4,5^, and it can occur in any part of the gastrointestinal tract, with the most common sites being the stomach (60–65%), followed by the small intestine (20–25%), and less commonly in the colorectum, esophagus and other locations^6,7^. Currently, surgical resection remains the preferred method for treating GIST. GIST rarely invade adjacent structures and lymph nodes; therefore, there is no need to expand the surgical margin or perform lymph node dissection. In recent years, with the rapid development of endoscopic technology, endoscopic resection has emerged as a new option for GIST treatment. Compared to surgical procedures, endoscopic resection is associated with less trauma, lower rates of complications, and shorter hospital stays^8,9^. While both domestic and international guidelines recommend surgical resection for GIST larger than 5 cm, there are still studies discussing the effectiveness and safety of endoscopic treatment for GIST larger than 5 cm^10–12^. At present, there is a lack of research focusing on the clinical question of whether endoscopic treatment is safe and effective for GIST larger than 5 cm. Therefore, the aim of this study is to explore the long-term survival outcomes of endoscopic treatment compared to surgical treatment for GIST in the range of 5–10 cm. Considering the low incidence of GIST, we chose to conduct this study using the Surveillance, Epidemiology, and End Results (SEER) database in the United States.

## Methods

### Population selection

**Figure 1:**
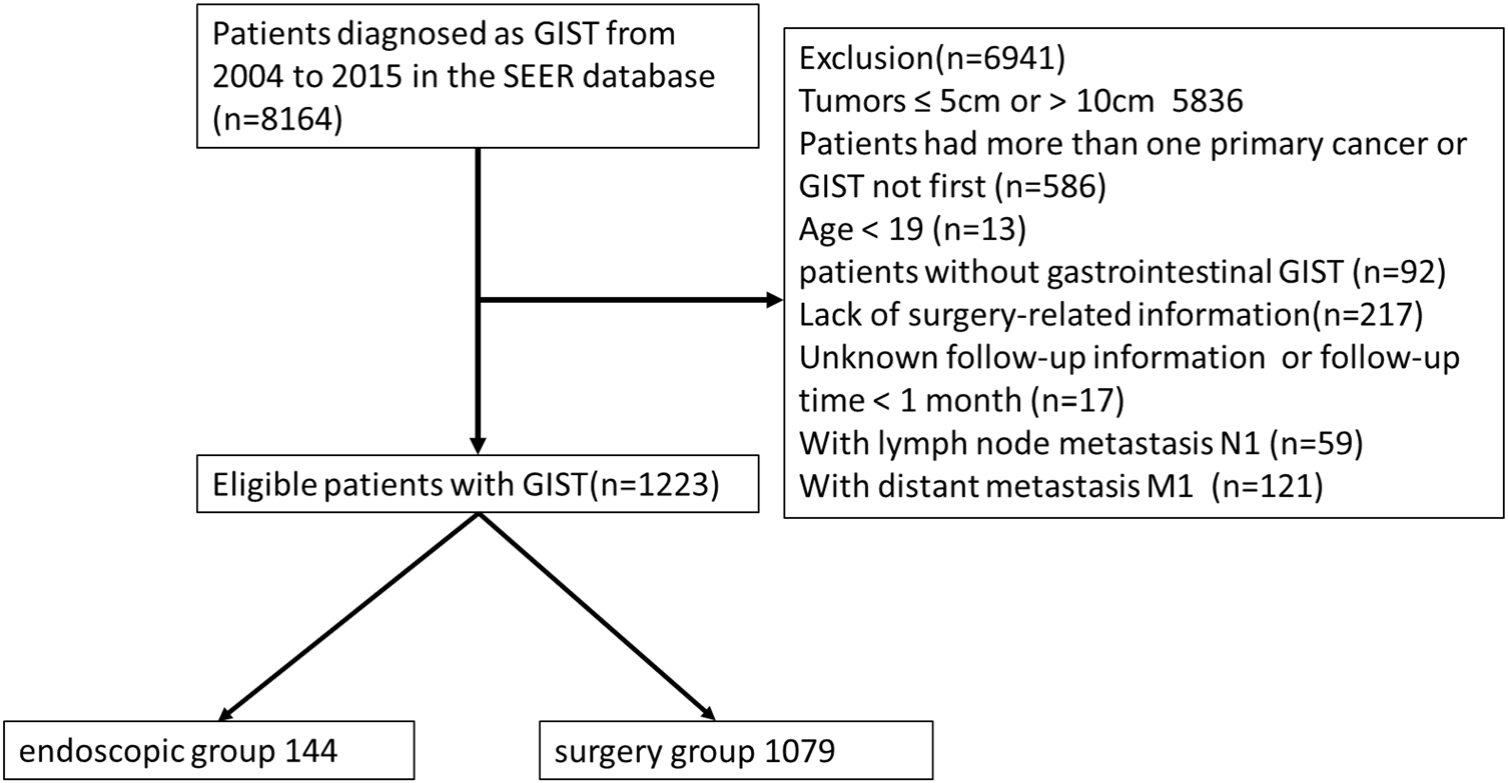
Flow chart of eligible patients diagnosed with 5–10 cm GIST.

Based on data collected from the SEER database, we conducted an analysis of the survival outcomes of GIST patients who underwent endoscopic and surgical treatments from 2004 to 2015. The SEER program is a project of the National Cancer Institute (NCI) in the United States, encompassing demographic information, tumor characteristics, tumor-related treatment details, and covering approximately 30% of cancer cases in the United States (https://seer.cancer.gov/). SEER*Stat software (version 8.4.0) was utilized to identify patients diagnosed with GIST in the SEER database between 2004 and 2015. According to the third edition of the International Classification of Diseases for Oncology (ICD-O-3), the specific histological code for GIST is 8936. We set up the exclusion criteria for this study: (1) Tumor size ≤ 5 cm or > 10 cm; (2) Patients had more than one malignant tumor; (3) Age < 19; (4) patients without gastrointestinal GIST; (5) Lack of surgery-related information; (6) Unknown follow-up information or follow-up time < 1 month; (7) With lymph node metastasis or distant metastasis. Finally, 1223 patients were selected for our study. A flow chart of the screening process is displayed in Fig. 1.

### Covariates and outcomes

We collected relevant patient data from the SEER database, including demographic information (age, sex, race, year of diagnosis and marital status), tumor characteristics (tumor size, tumor location, mitotic rate, T stage, N stage, M stage, tumor grade), chemotherapy, survival time, cause of death and treatment methods. According to the SEER Program Coding and Staging Manual, the treatment methods for GIST are categorized into endoscopic resection (ER) (codes: 20–27) and surgical resection (SR) (codes: 30–80). Additionally, we divided patient age into two groups: < 60 years and ≥ 60 years. Furthermore, using X-Tile software version 3.6.1 to determine the optimal cut-off value for tumor size, we categorized tumor size into two groups: ≤ 74 mm and 75–100 mm (Supplementary Fig. S1). The primary outcomes of the study are the overall survival (OS) and cancer-specific survival (CSS) of GIST patients undergoing endoscopic or surgical treatment. Accordingly, OS and CSS were calculated based on the date of diagnosis until the date of GIST-related death or the last follow-up date.

### Ethical approval and consent to participate

We confirm that all methods were carried out in accordance with relevant guidelines. The data for this study were obtained from the SEER database. Sample collection, research design was approved by the Ethics Committee of The First Affiliated Hospital of Nanchang University. We confirm that informed consent was obtained from all subjects and/or their legal guardian(s).

### Statistical analysis

Based on different treatment methods, patients were divided into two groups: the endoscopic treatment group and the surgical treatment group. For continuous data, if the data in both groups met the assumptions of normality and homogeneity of variance, we used the t-test for between-group comparisons; otherwise, we considered using the non-parametric Wilcoxon rank-sum test. For the comparison of categorical data, we employed the chi-square test or Fisher’s exact test. A 1:1 propensity score matching (PSM) was conducted to adjust for the distribution of patients between the two groups, with a caliper value set at 0.05. Covariates applied to the logistic regression model included age, sex, race, marital status, year of diagnosis, tumor site, tumor size, pathological differentiation grade, mitotic rate and chemotherapy. After PSM, we re-evaluated the clinical and pathological characteristics of the two patient groups. In this study, the Kaplan–Meier method was utilized to describe the survival changes in both groups, and the log-rank test was employed to compare differences in survival curves. The median follow-up time was calculated using the reverse Kaplan–Meier method. Cox regression analysis was used to adjust for other confounding factors, and hazard ratios (HRs) with 95% confidence intervals (CIs) were calculated to summarize the results. All data analyses were conducted using R software version 4.3.1. Survival curves were generated using the “survminer” and “survival” packages in R, the “MachIt” package was used to execute the PSM process. The software packages used in this manuscript were obtained from the website (https://www.r-project.org/). All *P*-values were two-tailed, and results were considered statistically significant when* P* < 0.05.

## Results

### Patient characteristics

According to the inclusion and exclusion criteria, this study analyzed a total of 1223 gastrointestinal GIST patients (617 males, 50.5%; 606 females, 49.5%). The clinicopathological characteristics of the two groups of patients are presented in Table 1. Among them, 144 patients underwent endoscopic treatment, and 1079 patients underwent surgical treatment. Of the total, 44.6% for age < 60 years old and 55.4% for age ≥ 60 years old. When comparing baseline characteristics between the two groups, we found no significant differences in age, sex, race, marital status, tumor site, tumor size and pathological differentiation grade (*P* > 0.05). In comparison to the endoscopic group, the surgical group tended to have a higher mitotic rate (> 5/50, 9.7% vs. 14.6%; *P* < 0.031). Patients diagnosed with GIST in the years 2012–2015 were more inclined to undergo surgical treatment (25.7% vs. 37.0%; *P* = 0.01). Those receiving surgical treatment were more likely to received chemotherapy (34.7% vs. 46.6%; *P* = 0.009).

**Table 1:** Comparison of baseline characteristics between endoscopic group and surgery group in 5–10 cm GIST patients before and after PSM.

Variables	Before PSM			After PSM		
	Endoscopic group	surgical group	***p*** value	Endoscopic group	surgical group	***p*** value
	n = 144	n = 1079		n = 144	n = 144	
Age, %			0.302			0.124
< 60	58 (40.3)	488 (45.2)		58 (40.3)	72 (50.0)	
≥ 60	86 (59.7)	591 (54.8)		86 (59.7)	72 (50.0)	
Sex, %			1			1
Male	73 (50.7)	544 (50.4)		73 (50.7)	74 (51.4)	
Female	71 (49.3)	535 (49.6)		71 (49.3)	70 (48.6)	
Race, %			0.173			0.424
White	106 (73.6)	716 (66.4)		106 (73.6)	110 (76.4)	
Black	21 (14.6)	177 (16.4)		21 (14.6)	14 (9.7)	
Other	17 (11.8)	186 (17.2)		17 (11.8)	20 (13.9)	
Marital, %			0.22			0.947
Married	122 (84.7)	851 (78.9)		122 (84.7)	122 (84.7)	
Unmarried	16 (11.1)	180 (16.7)		16 (11.1)	15 (10.4)	
Unknow	6 (4.2)	48 (4.4)		6 (4.2)	7 (4.9)	
Year of diagnosis, %			0.01			0.817
2004–2007	54 (37.5)	295 (27.3)		54 (37.5)	58 (40.3)	
2008–2011	53 (36.8)	385 (35.7)		53 (36.8)	48 (33.3)	
2012–2015	37 (25.7)	399 (37.0)		37 (25.7)	38 (26.4)	
Tumor site, %			0.408			0.554
Gastric	81 (56.2)	650 (60.2)		81 (56.2)	75 (52.1)	
Non-gastric	63 (43.8)	429 (39.8)		63 (43.8)	69 (47.9)	
Tumor size, mm			0.924			0.692
median (IQR)	70.00 [60.00, 85.50]	70.00 [60.00, 85.00]		70.00 [60.00, 85.50]	70.00 [60.00, 85.00]	
Tumor size, mm, %			0.532			0.905
51–74	85 (59.0)	603 (55.9)		85 (59.0)	83 (57.6)	
75–100	59 (41.0)	476 (44.1)		59 (41.0)	61 (42.4)	
Grade (differentiated), %			0.214			0.291
Well/moderately	35 (24.3)	287 (26.6)		35 (24.3)	38 (26.4)	
Poorly/undifferentiated	10 (6.9)	119 (11.0)		10 (6.9)	17 (11.8)	
Unknow	99 (68.8)	673 (62.4)		99 (68.8)	89 (61.8)	
Mitotic rate, HPF, %			0.031			0.422
≤ 5/50	44 (30.6)	399 (37.0)		44 (30.6)	39 (27.1)	
> 5/50	14 (9.7)	158 (14.6)		14 (9.7)	21 (14.6)	
Unknow	86 (59.7)	522 (48.4)		86 (59.7)	84 (58.3)	
Chemotherapy, %			0.009			0.902
No	94 (65.3)	576 (53.4)		94 (65.3)	92 (63.9)	
Yes	50 (34.7)	503 (46.6)		50 (34.7)	52 (36.1)	

### Comparison of endoscopic and surgical outcomes

**Figure 2:**
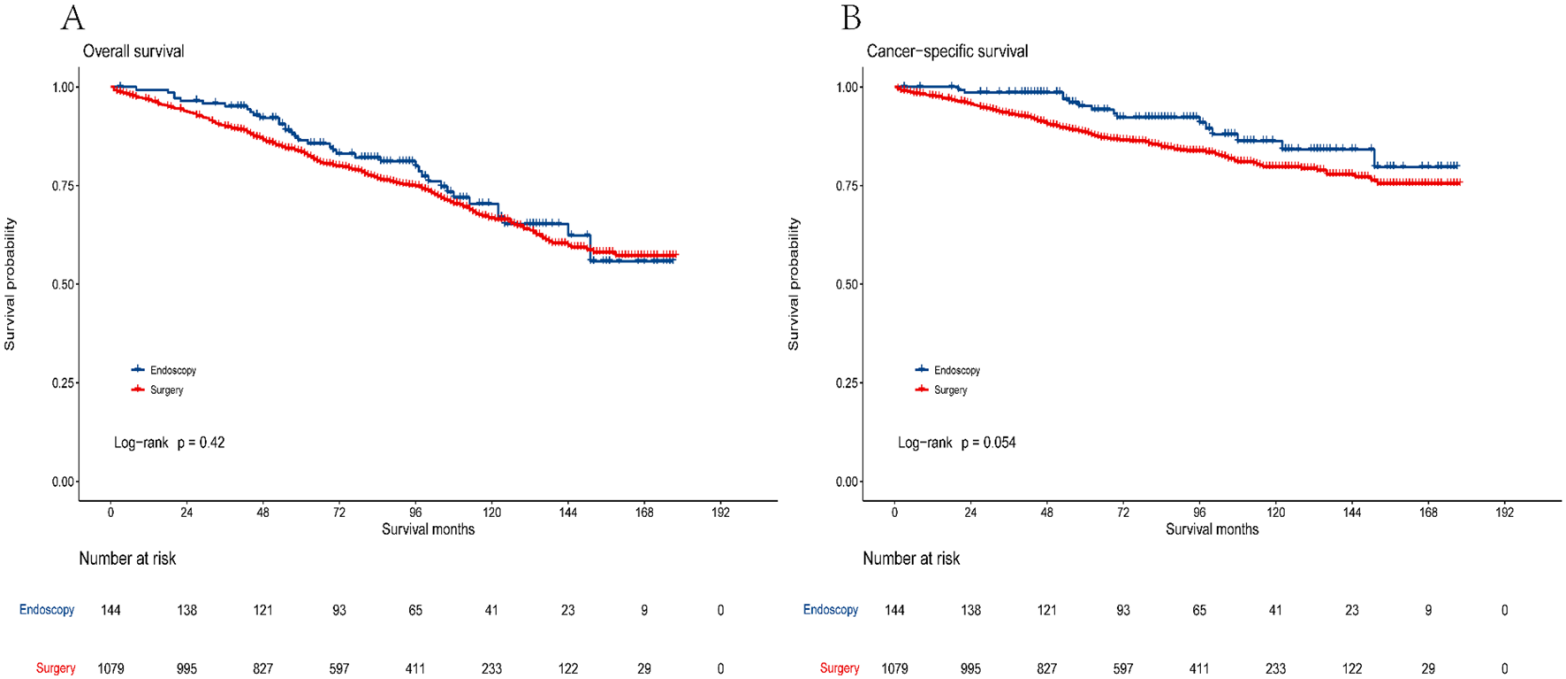
Kaplan–Meier curves of overall survival (**A**) and cancer-specific survival (**B**) according to treatment methods before PSM.

Before and after PSM, we conducted survival analysis and log-rank test. In this study, there were 38 deaths (26.4%) in the endoscopic treatment group and 288 deaths (26.7%) in the surgical treatment group. Among them, there were 15 patients in the endoscopic group and 168 patients in the surgical group who died from GIST. Additionally, we compared the survival outcomes of the two groups. The median follow-up time was 97 months for OS and 91 months for CSS. In these patients, the median survival time was not reached. The OS and CSS of the endoscopic group were comparable to the surgical group [5-year OS (86.5% vs. 83.5%, *P* = 0.42), 10-year OS (70.4% vs. 66.7%, *P* = 0.42), 5-year CSS (95.2% vs. 88.5%,* P* = 0.054), 10-year CSS (86.2% vs. 79.7%, *P* = 0.054)]. The survival curves for the two groups are shown in Fig. 2.

### Multivariable predictors of survival outcomes

The results of the multivariate Cox analysis are presented in Table 2. According to the results of the Cox regression model, we observed that the risk of OS (HR = 1.26, 95% CI 0.89–1.77, *P* = 0.19) and CSS (HR = 1.69, 95% CI 0.99–2.89, *P* = 0.053) was similar between the endoscopic treatment group and the surgical treatment group. Additionally, we found that older age (≥ 60 years old), female, tumor size ≥ 75 mm, poor/undifferentiated grade and a higher mitotic rate (> 5/50 HPF) were risk factors associated with decreased OS and CSS. Non-gastric was also identified as a risk factor for declining CSS but was not associated with patients OS. In the Cox analysis, race, marital status, year of diagnosis and chemotherapy showed no significant impact on the OS and CSS risks for patients.

**Table 2:** Multivariate cox regression analysis of OS and CSS in patients with 5–10 cm GIST.

Variables	OS	***p*** value	CSS	***p*** value
	HR (95% CI)		HR (95% CI)	
Age				
< 60	Reference		Reference	
≥ 60	3.11 (2.4–4.03)	< 0.001	2.01 (1.47–2.76)	< 0.001
Sex				
Male	Reference		Reference	
Female	0.66 (0.53–0.82)	< 0.001	0.61 (0.45–0.82)	0.001
Race				
White	Reference		Reference	
Black	1.33 (0.98–1.79)	0.066	1.18 (0.78–1.81)	0.432
Other	0.81 (0.58–1.11)	0.189	0.86 (0.56–1.31)	0.481
Marital status				
Married	Reference		Reference	
Unmarried	1.16 (0.84–1.59)	0.373	1.41 (0.96–2.08)	0.083
Unknow	1.04 (0.59–1.82)	0.897	0.99 (0.46–2.14)	0.984
Year of diagnosis				
2004–2007	Reference		Reference	
2008–2011	0.88 (0.66–1.17)	0.378	0.97 (0.66–1.43)	0.892
2012–2015	1 (0.66–1.5)	0.996	1.15 (0.68–1.94)	0.6
Tumor site				
Gastric	Reference		Reference	
Non-gastric	1.24 (0.99–1.56)	0.065	1.52 (1.12–2.05)	0.007
Tumor size, mm				
51–74	Reference		Reference	
75–100	1.35 (1.09–1.69)	0.007	1.42 (1.05–1.9)	0.021
Treatment				
Endoscopy	Reference		Reference	
Surgery	1.26 (0.89–1.77)	0.19	1.69 (0.99–2.89)	0.053
Grade (differentiated)				
Well/moderately	Reference		Reference	
Poorly/undifferentiated	2.21 (1.47–3.31)	< 0.001	2.44 (1.45–4.11)	0.001
Unknow	1.35 (0.99–1.85)	0.058	1.41 (0.92–2.17)	0.118
Mitotic rate, HPF				
≤ 5/50	Reference		Reference	
> 5/50	2.24 (1.51–3.31)	< 0.001	3.71 (2.2–6.23)	< 0.001
Unknow	1.49 (1.03–2.15)	0.032	2.25 (1.34–3.76)	0.002
Chemotherapy				
No	Reference		Reference	
Yes	0.97 (0.77–1.23)	0.793	1.12 (0.82–1.53)	0.465

### Propensity score matching

**Figure 3:**
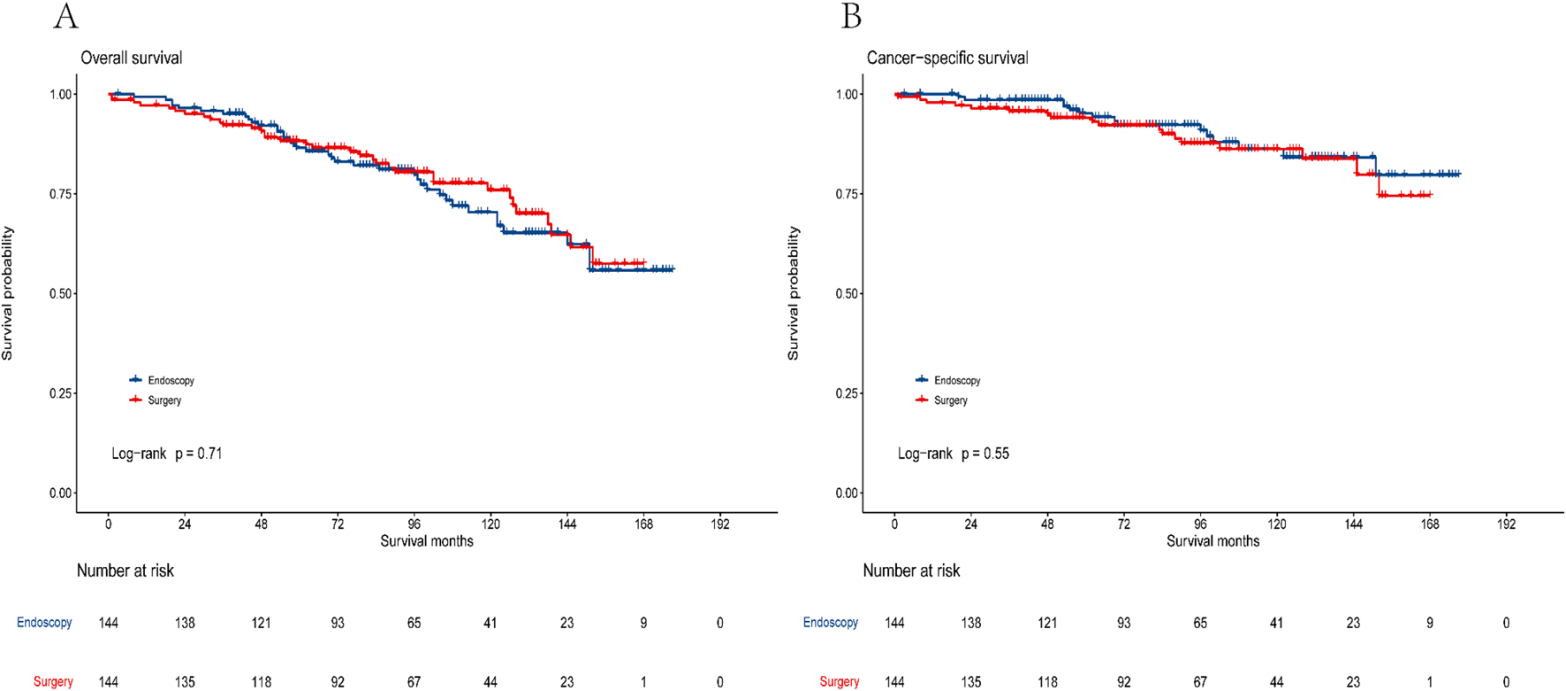
Kaplan–Meier curves of overall survival (**A**) and cancer-specific survival (**B**) according to treatment methods after PSM.

To eliminate differences between the two patient groups, we performed PSM with a caliper value of 0.05 to match all variables in a 1:1 ratio. After PSM, a total of 288 patients were generated, and as shown in Table 1, there were no significant differences in baseline characteristics between the endoscopic group (n = 144) and the surgical group (n = 144). The PSM histogram displayed a good distribution between the two groups after PSM (Supplementary Fig. S2). We further conducted survival analysis and log-rank tests after PSM to compare the survival outcomes of the two groups. There were no significant differences in the long-term survival rates between the endoscopic group and the surgical group [5-year OS (86.5% vs. 88.3%,* P* = 0.71), 10-year OS (70.4% vs. 76.0%, *P* = 0.71), 5-year CSS (95.2% vs. 94.1%, *P* = 0.55), 10-year CSS (86.2% vs. 86.2%, *P* = 0.55)]. The survival curves for the two groups post-PSM are shown in Fig. 3. Multivariate Cox regression analysis was conducted after PSM. Compared to the endoscopic group, the surgical group did not show a survival advantage in terms of OS (HR = 0.89, 95% CI 0.54–1.47, *P* = 0.649) and CSS (HR = 1.03, 95% CI 0.49–2.17, *P* = 0.937). After adjusting for confounding variables, we found that older age (HR = 2.66, 95% CI 1.51–4.67, *P* = 0.001) was an adverse prognostic factor for OS. Additionally, poorly/undifferentiated grade was identified as a risk factor for reducing both OS and CSS in patients (Supplementary Table S1).

## Discussion

Currently, surgery is the preferred method for treating 5–10 cm GIST. With the progress and widespread use of endoscopic techniques, a limited number of studies have suggested the feasibility of endoscopic treatment for large GIST^12,13^. However, there is scarce research comparing the safety and long-term efficacy of endoscopic and surgical treatments specifically for 5–10 cm GIST. Therefore, this study analyzed patients with 5–10 cm non-metastatic GIST from the SEER database, comparing the long-term outcomes of the two groups to address this question. Our survival analysis indicated that both before and after PSM, surgery did not show a survival advantage compared to endoscopic treatment. For patients with 5–10 cm GIST, independent prognostic factors predicting OS and CSS included age, sex, tumor size, pathological grade and mitotic rate. In our statistical analysis, surgically treated patients had a higher mitotic rate. Adjusting for covariates using a multivariate Cox model, we found that the impact of the two treatment methods on patient prognosis was similar. Furthermore, we utilized PSM to control for potential confounding factors and variable biases in this study. After PSM, we obtained two well-matched groups of patients and found no significant difference in the impact of the two treatment methods on patient prognosis. Overall, these findings suggest that endoscopic treatment for large 5–10 cm GIST is a safe and feasible option.

Tumor size is a crucial factor influencing the prognosis of GIST. Previous studies have indicated that endoscopic resection is considered safe and effective for GIST with a diameter smaller than 5 cm^14–16^. We utilized X-tile software to reclassify tumor sizes into two groups: 51–74 mm and 75–100 mm. Our multivariate Cox regression analysis revealed that a tumor size > 74 mm is a risk factor associated with decreased OS and CSS, this may be attributed to the larger the tumor, the higher the mitotic rate, increasing the risk of recurrence and metastasis^17^. Due to the substantial diameter of the tumor, complete endoscopic resection becomes challenging, raising the risk of positive margins. Simultaneously, related research suggests that tumor recurrence is influenced by the biological behavior of the tumor itself, rather than microscopic surgical margins^18,19^. Given these characteristics of GIST, endoscopic local resection has become a feasible option for treating GIST. A recent study has reported the technical feasibility of endoscopic resection for gastric GIST larger than 5 cm^12^. In this study, there were 18 patients who underwent endoscopic treatment and 63 patients who underwent surgical treatment, endoscopic treatment demonstrated similar short-term outcomes compared to laparoscopic treatment, with additional advantages such as rapid postoperative recovery and lower costs. Consistent with our research findings, the long-term survival outcomes of patients with GIST larger than 5 cm who underwent endoscopic treatment did not significantly differ from those who underwent surgical treatment.

Laparoscopic and endoscopic cooperative surgery (LECS) has been employed in the treatment of GIST^20^. LECS combines these two cutting-edge technologies, enhancing efficacy while overcoming individual limitations. Compared to traditional surgery, LECS is less invasive; in contrast to simple endoscopic resection, LECS offers advantages such as precise localization and a high rate of complete lesion removal. However, classical LECS carries the potential risk of gastric content or tumor cell spillage into the abdominal cavity, as it requires opening the gastric wall during surgery, increasing the risk of abdominal inflammation and incision infection^21^. A recent single-center retrospective study analyzed clinical data from 23 cases (18 GIST cases) of endoscopic treatment of giant gastric subepithelial tumors without laparoscopic assistance^22^. The results showed that 22 cases (95.7%) were completely resection, with four patients experiencing complications, all of whom recovered after conservative treatment. During the follow-up period, no residual tumors or recurrences were observed, indicating that endoscopic treatment of giant gastric GIST without laparoscopic assistance is feasible.

Our study has some limitations that could introduce biases to the results. Firstly, the study population is derived from the SEER database, and we lack access to certain critical parameters such as complications, margin status, length of hospital stay, recurrence details and specifics regarding the techniques used in endoscopy and surgery. These factors could potentially impact the study outcomes. Secondly, GIST are relatively rare, and despite obtaining data from the SEER database for the years 2004 to 2015, with 144 patients undergoing endoscopic treatment, the sample size is relatively small, which might affect the analytical results. Finally, being a retrospective study, there is an inherent risk of selection bias, although we employed PSM and multivariate Cox models to control for confounding factors, there might still be some unaccounted variables influencing the analysis outcomes. Despite these limitations, our study also possesses notable strengths. We benefit from a relatively long follow-up duration, enabling the first-ever comparison of endoscopic treatment with surgical treatment for the long-term survival outcomes of 5–10 cm GIST. This study represents the most extensive evaluation to date of the management of 5–10 cm GIST with endoscopic treatment. We strictly adhered to inclusion and exclusion criteria and employed PSM to match the two groups, aiming to eliminate differences that could potentially bias our study. This research can aid clinicians in making decisions in clinical practice.

## Conclusion

In summary, our study indicates that the long-term survival outcomes for patients with 5–10 cm GISTs are similar between the endoscopic and surgical groups. However, considering the limitations of our study, future prospective multicenter collaborative research is needed to validate our findings.

### Supplementary Information

Supplementary Figures.

Supplementary Table 1.

## Data Availability

The data presented in this study are available on request from the corresponding authors (CYZ and YXC). This data can be found here: https://seer.cancer.gov/.
