# Supplementary material for: Comparable long‑term survival outcomes of endoscopic treatment versus surgical treatment for gastrointestinal stromal tumors with a diameter of 5–10 cm
Source: Sci Rep. 2024 Apr 12;14:8513. doi: 10.1038/s41598-024-58802-4 (PMC11014986; doi:10.1038/s41598-024-58802-4)
Supplement: Supplementary file 1 — Supplementary Figures. [file 41598_2024_58802_MOESM1_ESM.pdf]

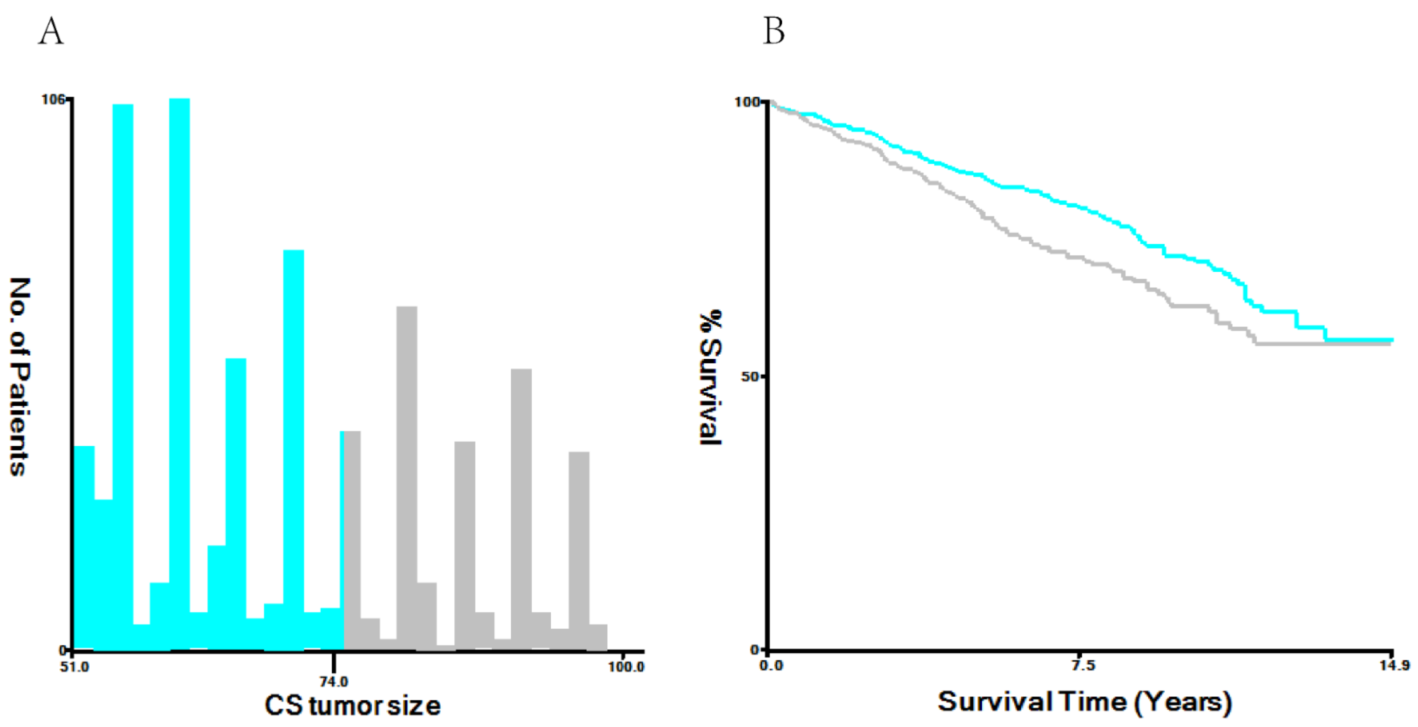

Supplement Fig 1. Optimal cut off values for tumor size based on X-tile.

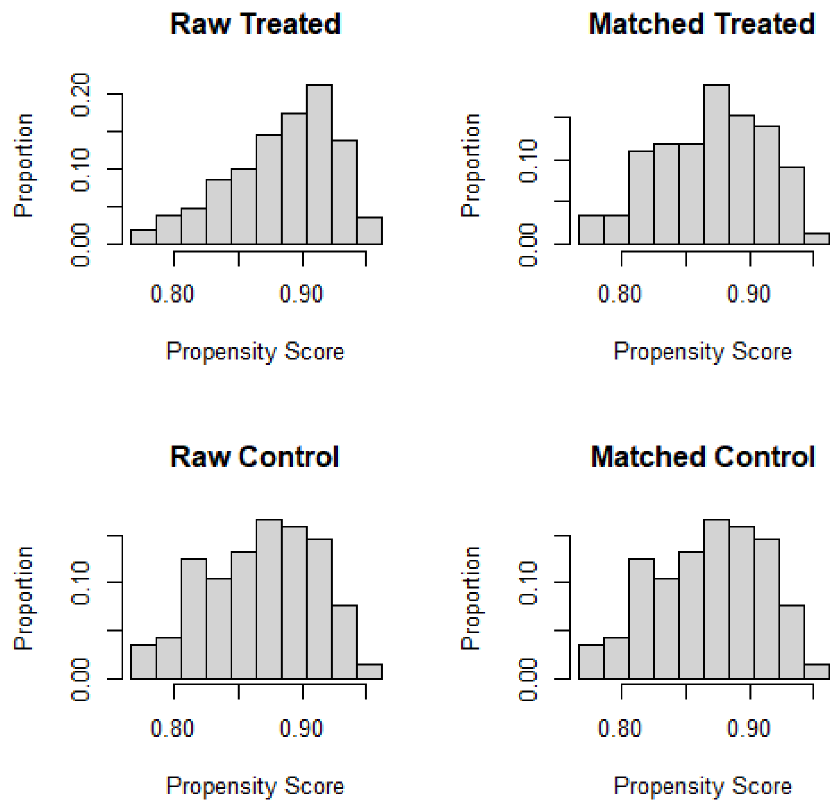

Supplement Fig 2. Histogram of PSM.
