# Supplementary material for: Comparable long‑term survival outcomes of endoscopic treatment versus surgical treatment for gastrointestinal stromal tumors with a diameter of 5–10 cm
Source: Sci Rep. 2024 Apr 12;14:8513. doi: 10.1038/s41598-024-58802-4 (PMC11014986; doi:10.1038/s41598-024-58802-4)
Supplement: Supplementary file 2 — Supplementary Table 1. [file 41598_2024_58802_MOESM2_ESM.docx]

Supplement Table 1 Multivariate cox regression analysis of OS and CSS in patients with 5-10 cm GIST after PSM.

| Variables | OS | ***p*** value | CSS | ***p*** value |
| --- | --- | --- | --- | --- |
|  | HR(95%CI) |  | HR(95%CI) |  |
| Age |  |  |  |  |
| <60 | reference |  | reference |  |
| ≥60 | 2.66(1.51-4.67) | 0.001 | 1.74 (0.82-3.72) | 0.152 |
| Sex |  |  |  |  |
| Male | reference |  | reference |  |
| Female | 0.62(0.37-1.01) | 0.057 | 0.72 (0.34-1.52) | 0.389 |
| Race |  |  |  |  |
| White | reference |  | reference |  |
| Black | 1.07(0.54-2.13) | 0.845 | 0.47 (0.11-2.1) | 0.324 |
| Other | 1.07(0.52-2.21) | 0.846 | 1.16 (0.41-3.29) | 0.78 |
| Marital status |  |  |  |  |
| Married | reference |  | reference |  |
| Unmarried | 1.18(0.48-2.87) | 0.72 | 1.42 (0.46-4.37) | 0.546 |
| Unknow | 2.17(0.83-5.68) | 0.115 | 0.82 (0.11-6.35) | 0.847 |
| Year of diagnosis | |  |  |  |
| 2004-2007 | reference |  | reference |  |
| 2008-2011 | 0.67(0.34-1.34) | 0.26 | 0.82 (0.3-2.26) | 0.705 |
| 2012-2015 | 0.78(0.28-2.12) | 0.621 | 0.82 (0.19-3.5) | 0.786 |
| Tumor site |  |  |  |  |
| Gastric | reference |  | reference |  |
| Non-gastric | 0.88(0.52-1.47) | 0.621 | 1.43 (0.67-3.05) | 0.36 |
| Tumor size, mm |  |  |  |  |
| 51-74 | reference |  | reference |  |
| 75-100 | 1.26(0.77-2.07) | 0.364 | 1.78 (0.86-3.69) | 0.122 |
| Treatment |  |  |  |  |
| Endoscopy | reference |  | reference |  |
| Surgery | 0.89(0.54-1.47) | 0.649 | 1.03 (0.49-2.17) | 0.937 |
| Grade (differentiated) | |  |  |  |
| Well/moderately | reference |  | reference |  |
| Poorly/undifferentiated | 2.66(1.1-6.38) | 0.029 | 5.37 (1.3-22.13) | 0.02 |
| Unknow | 1.29(0.68-2.48) | 0.437 | 2.81 (0.82-9.58) | 0.099 |
| Mitotic rate, HPF |  |  |  |  |
| ≤5/50 | reference |  | reference |  |
| ＞5/50 | 2.44(0.93-6.43) | 0.071 | 3.31 (0.92-11.84) | 0.066 |
| Unknow | 1.59(0.66-3.81) | 0.303 | 1.18 (0.34-4.12) | 0.793 |
| Chemotherapy |  |  |  |  |
| No | reference |  | reference |  |
| Yes | 1.49(0.84-2.64) | 0.173 | 1.47 (0.65-3.31) | 0.352 |
